# New species of *Ceratostema* (Ericaceae, Vaccinieae) from the southeast Andes of Ecuador-II

**DOI:** 10.3897/BDJ.13.e158872

**Published:** 2025-08-19

**Authors:** Marco M. Jiménez, Gabriel A. Iturralde, Luis Ocupa-Horna, J. R. Kuethe, Henry X. Garzón-Suárez

**Affiliations:** 1 Grupo de Investigación en Biodiversidad, Medio Ambiente y Salud BIOMAS, Carrera de Ingeniería Agroindustrial, Facultad de Ingeniería y Ciencias Aplicadas, Universidad de Las Américas, UDLA, Vía a Nayón, Quito, Ecuador Grupo de Investigación en Biodiversidad, Medio Ambiente y Salud BIOMAS, Carrera de Ingeniería Agroindustrial, Facultad de Ingeniería y Ciencias Aplicadas, Universidad de Las Américas, UDLA, Vía a Nayón Quito Ecuador; 2 Herbario Pedro Ruiz Gallo (PRG), Universidad Nacional Pedro Ruiz Gallo, Calle Juan XXIII 391, Lambayeque, Peru Herbario Pedro Ruiz Gallo (PRG), Universidad Nacional Pedro Ruiz Gallo, Calle Juan XXIII 391 Lambayeque Peru; 3 Grupo Científico Calaway Dodson: Investigación y Conservación de Orquídeas del Ecuador, Quito, Ecuador Grupo Científico Calaway Dodson: Investigación y Conservación de Orquídeas del Ecuador Quito Ecuador; 4 School of Environment, Faculty of Sciences, University of Auckland, City Campus for Science and Engineering, Symonds Street, Auckland, New Zealand School of Environment, Faculty of Sciences, University of Auckland, City Campus for Science and Engineering, Symonds Street Auckland New Zealand; 5 Herbario HUTPL, Departamento de Ciencias Biológicas, Universidad Técnica Particular de Loja, Loja, Ecuador Herbario HUTPL, Departamento de Ciencias Biológicas, Universidad Técnica Particular de Loja Loja Ecuador; 6 Jungle Dave’s Science Foundation, San Juan Bosco, Ecuador Jungle Dave’s Science Foundation San Juan Bosco Ecuador

**Keywords:** Morona Santiago, northern Andes, submontane forest, south-eastern Ecuador, taxonomy

## Abstract

**Background:**

*Ceratostema* is a genus in the Ericaceae comprising, approximately 50 species and is distributed from Venezuela and Guyana to northern Peru in South America. The country with the greatest diversity of the genus is Ecuador, where most taxa are restricted to the submontane and montane forests of the eastern slopes of the Andes. The "*auriculatum* group" refers to a set of species restricted to south-eastern Ecuador, characterised by plants with an epiphytic and pendant habit, amplexicaul leaves that partially enclose the flowers, few-flowered inflorescences provided with small floral bracts and bracteoles and a calyx with a non-prominent limb.

**New information:**

Two new species of *Ceratostema* from the Morona Santiago province, Ecuador, belonging to the informal morphological “*auriculatum*” group are described and illustrated here. *Ceratostemagearyana* has plinerved, suborbicular leaves that are pruinose with lobes that do not overlap at the base; the mid-vein that is raised throughout its length; and a calyx with an obconic hypanthium with deltate-ovate lobes. It is compared with *C.cutucuense* from which it is distinguished by the smaller leaves, the glabrous floral parts, the hypanthium smaller, obconic, obscurely pentagonal, the larger calyx lobes and the corolla with narrowly linear-triangular lobes. *Ceratostemamoronasantiagoensis* has pinnately veined, ovate leaves, with basally imbricate lobes, deltate calyx lobes and a fuchsia-red, pubescent corolla with puberulous stamens. It is most similar to *C.auriculatum*, but it is distinguished by the calyx limb unlobed at the base, the smaller, acuminate calyx lobes, not conspicuously nerved, pubescent overall, the fuchsia red, pubescent corolla, with longer lobes, and the puberulent, longer filaments. The taxonomic affinities of the two new species are discussed, and information about their distribution, habitat and conservation status is provided.

## Introduction

*Ceratostema* Juss. was first proposed by Jussieu (1789), using two terms derived from ancient Greek “*keratos*” and “*stema*” meaning “*horn-like stamen*”, referring to the long anther tubules of the stamens (Luteyn 1986, Quattrocchi 2000). The genus comprised 39 recognised species (Cornejo and Luteyn 2024), nine of which were discovered in the last few years (Cornejo and Luteyn 2024, Doucette et al. 2024, Doucette et al. 2025a, Doucette et al. 2025b, Doucette et al. 2025c, Doucette et al. 2025d). In addition to the two new species described here, the total number of species in the genus is 50.

Pedraza-Peñalosa et al. (2015), based on a phylogenetic analysis using nuclear (nrITS) and plastid (*ndhF*, *matK*) markers, recovered *Ceratostema* as a non-monophyletic genus within the Andean Clade B of Vaccinieae. Their results place *Ceratostema* in a poorly-resolved tritomy together with *Psammisia* I and the clade *Psammisia* II + *Macleania*, forming what the authors refer to as the *Psammisia–Ceratostema–Macleania* clade. Notably, *Ceratostemareginaldii* (Sleumer) A.C.Sm. was nested within *Macleania*, suggesting that some species currently assigned to *Ceratostema* are more closely related to *Macleania* or *Psammisia*. Although *Ceratostema* is generally recognized as morphologically distinct (Luteyn 2005, Jiménez et al. 2021, Cornejo et al. 2024, Jiménez et al. 2024), current phylogenetic inferences are based on limited taxon sampling, and additional data are needed to clarify its evolutionary relationships with related genera in Vaccinieae (Pedraza-Peñalosa et al. 2015).

*Ceratostema* is distributed across South America, from Venezuela and Guyana to as far south as northern Peru (Luteyn 2005, Luteyn 2021), where it ranges in elevation from 450 to 3950 m (Luteyn 1996). Thirty-six species, including the two described here, are known from Ecuador (Cornejo and Luteyn 2024), where most taxa are restricted to the submontane to montane forests on the eastern slopes of the Andes (Luteyn 1996, Jiménez et al. 2021, Jiménez et al. 2024). *Ceratostema* can be easily recognised by the articulated pedicels down to the calyx, the large corollas with proportionately elongated lobes, the stamens being as long as the corolla, but individually slightly unequal in length. Furthermore, the anthers have coarsely papillose thecae and the width of the elongated tubules is about half the diameter of the thecae (Luteyn 2005).

Luteyn (2005) mentions a distinct group of morphologically similar species of *Ceratostema* which share a set of unique features, including the epiphytic, long-pendant habit; the branches being embraced by amplexicaul, cordate and convex, pinnately-nerved leaves that are folded downwards from the base and, somewhat, hiding the few-flowered inflorescences. Moreover, to these traits we should add the axillary inflorescences themselves being sessile; with minute floral bracts and bracteoles and a 5-winged hypanthium at the calyx with a not prominent limb. Additionally, species exhibiting these traits appear to be geographically restricted to just the southeast of Ecuador. Thus far, species included in this morphological group, here informally referred to as the "*auriculatum* group", are: *C.auriculatum* Luteyn, *C.cutucuense* Luteyn, *C.macbrydiorum* Luteyn, *C.pendens* Luteyn and the recently described *C.ingridportillae* A. Doucette, H. Medina & J. Portilla (Luteyn 2005, Doucette et al. 2025c). Most recently, two more species belonging to this morphological group were discovered in south-eastern Ecuador and are described and illustrated in this paper.

## Materials and methods

Plant material of the new species was collected at the Agett-Geary Reserve, near San Juan Bosco, under research permit No. MAATEDB I-CM-2022-0248 endorsed by the Ministerio del Ambiente, Agua y Transición Ecológica. The Reserve covers an area of 312.4 hectares. According to the Ministerio del Ambiente del Ecuador (2013), the Reserve is situated within the evergreen premontane forest at the south-eastern Cordillera of the Andes (BsPn04). This region is characterised by a mean annual temperature of 21.6°C and average annual precipitation of approximately 1945 mm. The climate ranges from humid to perhumid with frequent fog during the rainy season. Vegetation in the area includes cattle pastures as well as patches of secondary and mature forests, the latter typically featuring trees up to 20 m in height. Dominant species include *Cecropia* sp., *Ficus* sp., *Dacryodesperuviana* (Loes.) H.J.Lam, *Erythrinapoeppigiana* (Walp.) O.F.Cook, *Ingaedulis* Mart., *Nectandra* sp. and *Wettiniamaynensis* Spruce. The forest also supports a rich abundance of epiphytic plants, notably aroids, bromeliads, bryophytes, ericads, gesneriads and orchids.

Digital images of the available specimens of *Ceratostema* stored at F, MO, NY, QCNE, U, and WIS Herbaria (Thiers 2025) were examined through iDigBio (https://portal.idigbio.org/portal/search) and the National Diversity Database of the INABIO (https://bndb.sisbioecuador.bio/bndb/). Vouchers identified as *Ceratostema* deposited at the LOJA Herbarium were examined physically to ensure there were no paratypes of the new species. Rarity and restricted access were limitations for finding additional specimens of the new species; only single specimens’ vouchers were available, which served as holotypes.

Photographs were taken using a Nikon® D3100 camera with AF-S DX Micro NIKKOR 40 mm f/2.8G lens complemented with two Nikon SB-700 AF speedlight flashes and a Panasonic® FZ300 camera with Raynox DCR-150 mm Super Macro lens. The figures and the composite plate were prepared with Adobe Photoshop v. 24.0.1 and 21.0.03. Fresh flowers were preserved in 70% ethanol and 1% glycerol. Dry material was deposited at the HUTPL Herbarium. All specialised literature concerning the genus *Ceratostema* and herbarium specimens of related and morphologically similar species were examined and compared with the material presented here (Luteyn 1996, Luteyn 2005, Doucette et al. 2025c). Digital images of the holotypes of *Ceratostemaauriculatum* and *C.cutucuense* stored at NY were examined through JSTOR Global Plants (htpps://plants.jstor.org). The new species are described following the botanical terminology defined by Beentje (2012).

## Taxon treatments

### Ceratostema gearyana

M.M.Jiménez & H.Garzón sp. nov.

2549F37C-73E7-5E8A-8593-0FB525CDA791

#### Materials

- **Type status:** Holotype. **Occurrence:** recordNumber: H. Garzón 295; recordedBy: H. Garzón; occurrenceID: 78A4C16D-A458-529E-AAF3-5B552693A512; **Taxon:** scientificName: *Ceratostemagearyana* M.M.Jiménez & H.Garzón; **Location:** country: Ecuador; stateProvince: Morona Santiago; locality: Reserva Agett-Geary, cerca de San Juan Bosco; verbatimElevation: 1663 m; **Event:** year: 2025; month: 5; day: 1; **Record Level:** institutionCode: HUTPL!

#### Description

**Figure 1.**
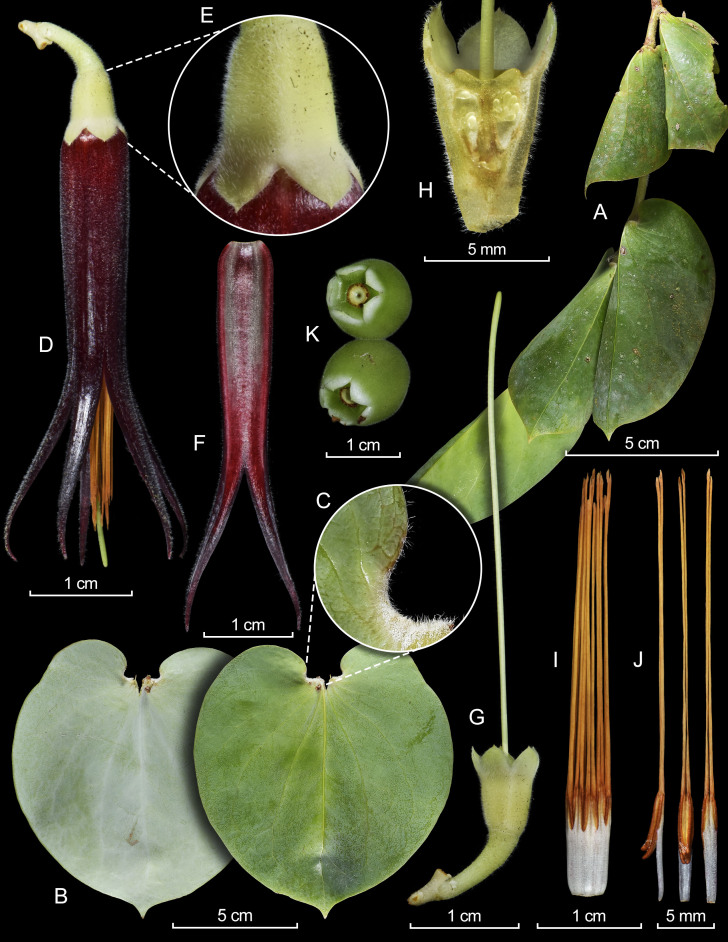
Lankester Composite Dissection Plate (LCDP) of *Ceratostemagearyana*. **A** Terminal branch; **B** Leaves in abaxial (left) and adaxial (right) views with a close-up of the basal margin **(C)**; **D** Flower with rachis and a close-up of the calyx lobes **(E)**; **F** Longitudinal section of the corolla; **G** Flower without corolla showing the ovary and style; **H** Longitudinal section of the calyx showing the ovary, hypanthium and limb; **I** Staminal tube; **J** Stamens, lateral (left), ventral (middle), dorsal (right) views; **K** Mature fruits. Prepared by L. Ocupa-Horna, based on photographs of the type by H.X. Garzón-Suárez.

*Shrubs* pendant, epiphytic; indumentum consisting of almost persistent trichomes, white, eglandular, 0.3–1.1 mm long, arranged unevenly, sparsely to densely on younger branches, petioles, leaf blades, inflorescences and flowers, excluding stamens; axonomorphous roots with a woody, subpsherical, well-developed lignotuber-like swelling. *Stems* terete to subterete, ca. up to 1.4 m long, glabrous, slightly arching, arising from the swelling, older stems dark brown, cracking longitudinally and exfoliating; younger branches terete, ca. 80 cm long, 2.1 mm wide, pendant, slightly arcuate, pale green, puberulous, becoming striate and dark brown when old or after exfoliation; axillary buds emerging 1 mm below the leaf node, axillary bracts 1.2–2.5 mm long, pale green, sparsely pubescent, ovate triangular. *Leaves* spirally arranged, pendulous; petioles pale green, subterete 3.8 mm × 2.1–2.3 mm, pubescent; blades dark green in older, paler in mature and mahogany in younger leaves, cordiform to ovate-orbicular, 10.3–12.8 cm × 6.6–8.6 cm, coriaceous, convex with basal margins folded to conceal flowers and fruits, glabrous adaxially, with very few sparse hairs at the basal margin abaxially, pruinose adaxially and abaxially, 7–9-plinerved from near the base, sometimes with an inner pair of lateral nerves arising from the proximal 0.8–1.5 cm, midvein raised along almost its length adaxially, impressed and hollow abaxially, secondary veins slightly raised adaxially, impressed abaxially, branching, anastomosing distally with reticulate veinlets weakly raised to obscure, base cordate, margin undulate, apex shortly acuminate to acuminate. *Inflorescence* axillary, a cincinnus with very compact internodes, pendulous, sessile, 2–3-flowered; rachis obconical, 3.7–4.4 mm long, 1.8 mm thick, puberulous, subverrucose, covered at the base by several bracts; bracts persistent, whitish-green, ovate, 0.9–1.1 mm × 1.2–1.6 mm, ciliate to the margin, acute to obtuse at the apex; floral bracts persistent, similar in colour and texture to the peduncle bracts, ovate, 0.8–1.1 × 0.8–1.2 mm, acute; pedicel articulate with the calyx, pale green, subterete, 7.6–8.4 mm long, 1.9–2.5 mm thick, puberulous; bracteoles persistent, 2, whitish-green, ovate-triangular, 0.6–0.8 mm × 0.7-0.8 mm, located near the base and opposite, long-ciliate along the margins, apex attenuate. *Flowers* pentamerous with pale green calyx and dark reddish-brown corolla, paler at the base; calyx 7.2–8.9 mm × 6.1–6.6 mm, puberulous; hypanthium obconical, obscurely pentagonal, 5.5–6.3 mm × 3.7–4.1 mm, truncate; limb erect, open, 3.2–3.6 mm × 6.1–6.6 mm; lobes 5, ovate-deltate, 2.0–2.7 mm × 2.8–3.0 mm, slightly convex, apiculate at the apex, sinuses acute. *Corolla* tubular, but slightly dilated to the base and narrowing distally, thick-carnose, bistratose, 4.2–4.4 cm long (including the lobes), 6.5 mm in diameter at the base, 5.4 mm in diameter at the throat, puberulent and bluntly 5-angled along its length externally, puberulous in the internal apical half; lobes 5, spreading, narrowly linear-triangular, 19.8–22.3 mm × 2.2–3.0 mm, lustrous, puberulent externally, glabrous internally, channelled and subverrucose internally, slightly incurved to the base, recurved and acuminate at the apex. *Stamens* 10, with white filaments, golden yellow tubules and darker thecae, nearly equalling the corolla in overall length, each pair unequal, 3.6–4.0 cm long; filaments connate forming a tubular staminal tube, 8.8–9.2 mm long, glabrous outside, visibly papillose inside; anthers 3.2–3.6 cm long, each pair of thecae unequal, prognathous, 6.2–6.6 mm long, conspicuously papillose; tubules distinct, slightly unequal, seemingly free, 2.7–3.0 cm long, glabrous, dehiscing by terminal pores, ca. 0.9 mm × 0.4 mm. *Style* green, exserted, 4.4 cm long, pilose, stigma truncated. Mature *fruit* yellowish-green, subglobose, ca. 14.0–16.8 mm × 15.0 mm in diameter, puberulous, with persistent calyx lobes. Seed not seen (Fig. 1).

#### Diagnosis

*Ceratostemagearyana* is distinguished from *C.cutucuense* by the smaller leaves, 10.3–12.8 cm × 6.6–8.6 cm (vs. 14.0–16.0 cm × 9.0–11.0 cm), the glabrous floral parts (vs. puberulous), the hypanthium smaller, obconic, obscurely pentagonal (vs. turbinate, bluntly 5-winged), the larger calyx lobes deltate-ovate, 2.0–2.7 mm long (vs. truncate, < 0.5 mm long) and the corolla bluntly 5-angled (vs. bluntly 5-winged), with longer, narrowly linear-triangular lobes, 19.8–22.3 mm long (vs. linear-lanceolate, 12.0–14.0 mm long).

#### Etymology

This species is named after the Agett-Geary Reserve, a recently created private protected area, where the new species was discovered.

#### Distribution

**Figure 2.**
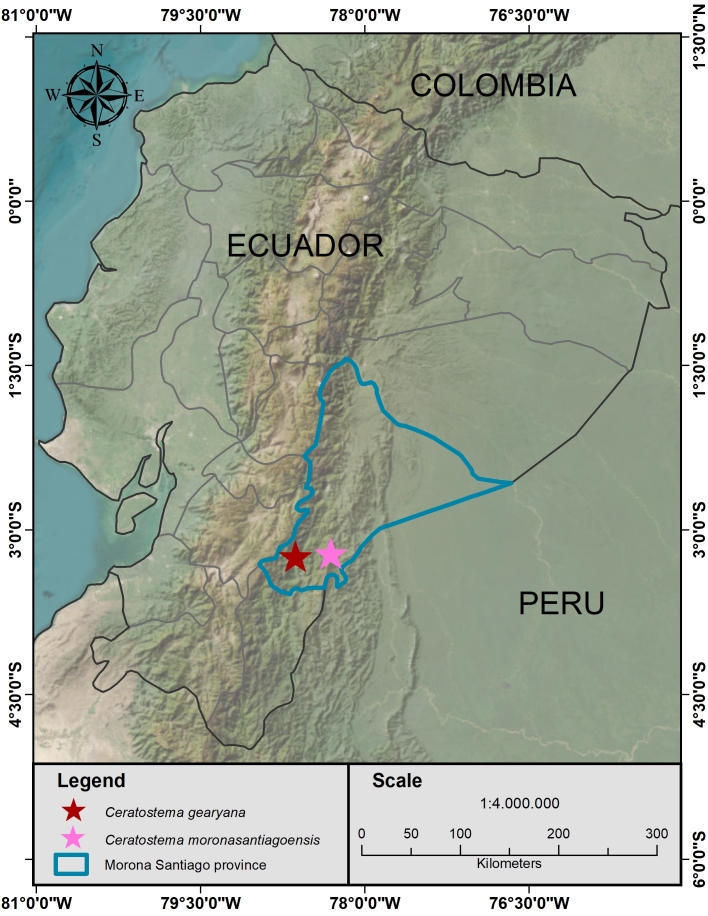
Distribution of *Ceratostemagearyana* and *C.moronasantiagoensis* in southern Ecuador.

*Ceratostemagearyana* is known from a single population near San Juan Bosco in the south-eastern Ecuadorian Province of Morona-Santiago (Fig. 2).

#### Ecology

**Figure 3.**
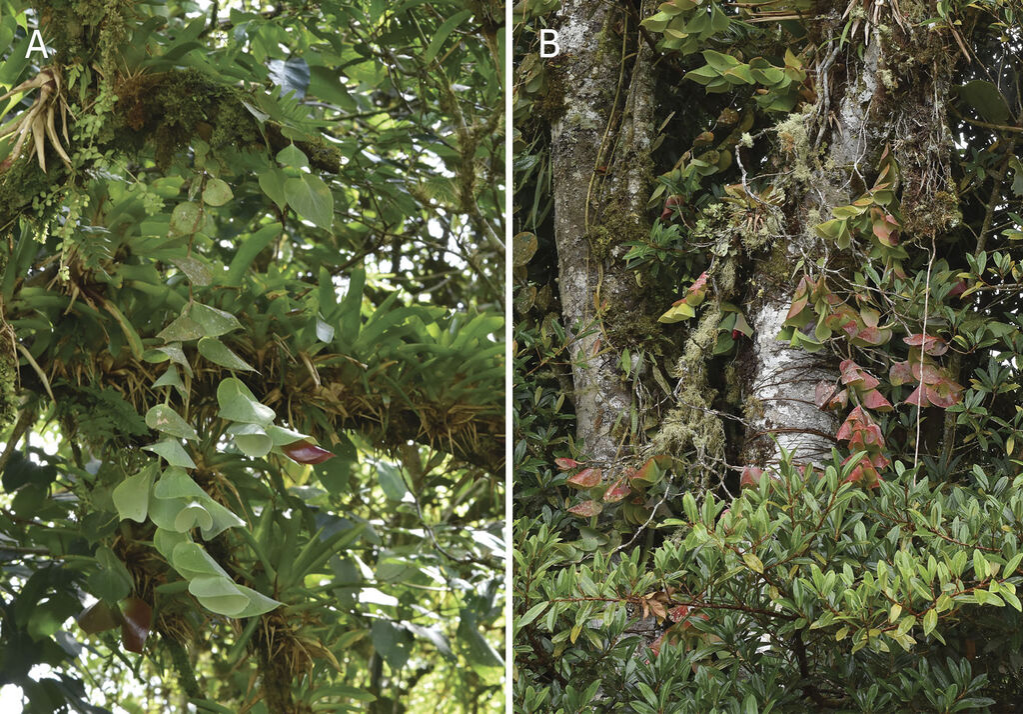
*Ceratostemagearyana* in situ. **A** Specimen hanging from a branch in the shade; **B** Specimens exposed to sunlight on tree trunks.

The new species grows as a pendant epiphytic species at elevations between 1400 to 1600 m (Fig. 3). This area is characterised by mature and disturbed primary forests where *C.gearyana* co-exists with other flora, including: *Blakeahirsutissima* (J.F. Macbr.) Wurdack, *Ceratostemaglans* Luteyn, *Elleanthusblatteus* Garay, *Sphyrospermumglutinosum* Luteyn & Pedraza and *S.kakabadseae* A. Doucette, H. Medina & J. Portilla.

#### Conservation

*Ceratostemagearyana* has been located within the buffer zone of the Siete Iglesias Municipal Conservation Area (ACMSI), but not inside the Reserve. Seven individuals are known from the type locality. The main threat to the conservation of this population remains the destruction of primary forests for timber, pasture and cattle grazing. Regardless, its proximity to the aforementioned municipal reserve, which is dedicated to the conservation of primary forest, led the authors to believe that *C.gearyana* is well represented within its boundaries and therefore protected.

Applying conservation analysis with the georeferenced single locality, the calculated area of occupancy (AOO) is 4 km^2^. The extent of occurrence (EOO) has not been calculated due to the limited number of populations sighted. For these reasons, the authors recommend *C.gearyana* should be considered Critically Endangered (CR) according to the IUCN Criteria B2ab(i,ii), C2a(i) and D1 (IUCN 2024).

#### Taxon discussion

*Ceratostemagearyana* is the newest addition to the informal "*auriculatum* group". Coherent traits for this group are: the epiphytic, long-pendant habit; the twigs are embraced by amplexicaul, cordate and convex, folded downwards at the base and hiding the few flowered inflorescences; the sessile, axillary inflorescences; the minute floral bracts and bracteoles; and the calyx with a 5-winged hypanthium and a non-prominent limb.

*Ceratostemagearyana* is most similar to *C.cutucuense* by the glaucous younger branches; the glabrous, suborbicular leaves; the erect calyx limb with the apiculate lobes; the acute sinuses; and the stamens having glabrous filaments. Nevertheless, *C.gearyana* is distinguished from *C.cutucuense* by the characters stated in the diagnosis. Further differences with the other species, additionally are the leaves that have basal lobes parallel to each other (vs. imbricate), the foliar apices being acuminate (vs. abruptly cuspidate to shortly acute), the 2–3-flowered inflorescences (vs. 4–5-flowered), the smaller pedicel, 7.6–8.4 mm long (vs. 12.0–13.0 mm long), the apically acuminate corolla lobes (vs. spurred), the stamens being comparatively shorter (3.6–4.0 cm long vs. ca. 5.0 cm long) and the filaments being comparatively longer (8.8–9.2 mm long vs. 6.0 mm long) (Luteyn 1996). The new species is further distinct within the “*auriculatum* group” by its notably pruinose leaves, where the basal margin becomes pubescent when mature and later glabrous at a post-mature stage.

### Ceratostema moronasantiagoensis

M.M.Jiménez & H.Garzón sp. nov.

D5FD71C0-4A43-52DE-9A6B-BE5F3D910A72

urn:lsid:ipni.org:names:77367755-1

#### Materials

- **Type status:** Holotype. **Occurrence:** recordNumber: H. Garzón 297; recordedBy: H. Garzón; occurrenceID: 4D149160-74F4-5EAD-8B32-DCB3753B4513; **Taxon:** scientificName: *Ceratostemamoronasantiagoensis* M.M.Jiménez & H.Garzón; **Location:** country: Ecuador; stateProvince: Morona Santiago; locality: Cerca de San Juan Bosco; verbatimElevation: 1492 m; **Event:** year: 2025; month: 5; day: 8; **Record Level:** institutionCode: HUTPL!

#### Description

**Figure 4.**
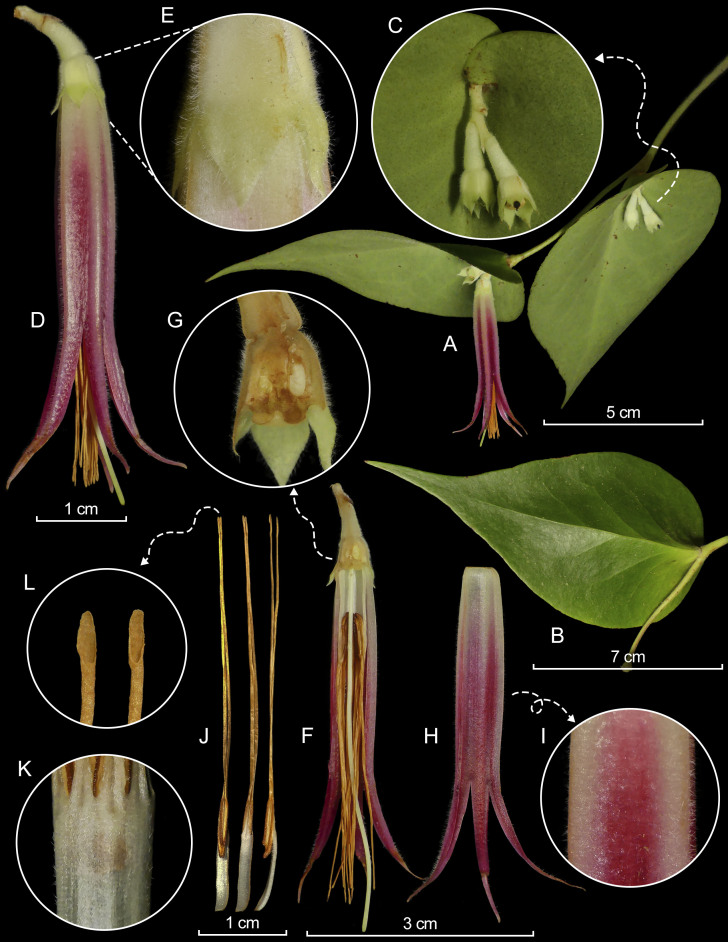
Lankester Composite Dissection Plate (LCDP) of *Ceratostemamoronasantiagoensis*. **A** Terminal branch with a close-up of the inflorescence **(C)**; **B** Leaf in adaxial view; **D** Flower with rachis and a close-up of the calyx **(E)**; **F** Longitudinal section of the flower with a close-up of the calyx **(G)**; **H** Longitudinal section of the corolla with close-up of the external surface **(I)**; **J** Stamens, ventral (left), dorsal (middle), lateral (right) views with a close-up of the terminal pores of the tubules **(L)**; **K** External surface of the staminal tube. Prepared by L. Ocupa-Horna based on photographs of the type by M.M. Jiménez.

*Shrubs* pendant, lianoid epiphytic; indumentum consisting of almost persistent trichomes, white, eglandular, 0.1–0.6 mm long, arranged unevenly, sparsely to densely on younger branches, petioles, underside of leaves, inflorescences and flowers, including stamens and style; axonomorphous roots with a woody, fusiform, well-developed lignotuber-like swelling, ca. 4.3–7.4 cm × 3.1–6.3 cm in diameter. *Stems* terete to subterete, ca. 52 cm long, glabrous, slightly arching, arising from the swelling, older stems dark brown, cracking longitudinally and exfoliating; younger branches subterete, ca. 51 cm long, 4.2 mm wide, descending to pendant, slightly arcuate, pale green, sometimes suffused with brownish-pink, pilose, becoming glabrous, striate and dark brown when old or after exfoliation; axillary buds emerging 1 mm below the leaf node. *Leaves* spirally arranged, pendulous to descending, new leaves mahogany red; petioles subterete, ca. 3.1 mm × 2.6 mm, pilose, becoming glabrous with age; blades dark green adaxially, pale green abaxially, ovate, convex, 6.8–14.3 cm × 4.5–6.9 cm, coriaceous, glabrous adaxially and abaxially, lustrous adaxially, dull abaxially, pinnately nerved with 3–4 lateral nerves per side, mid-vein raised along almost its length adaxially, impressed and hollow abaxially, the secondary veins slightly raised adaxially, impressed abaxially, branching, anastomosing distally with reticulate veinlets, obscure, base deeply cordate, partially folded downwards exposing the flowers, lobes auriculate, overlapping, apex acuminate to long-acuminate. *Inflorescence* axillary, a cincinnus with very compact internodes, 2–5-flowered, sessile; rachis obconical, ca. 9.9 mm long, 2.0–2.8 mm thick, covered at the base by several bracts; bracts persistent, pale green, ovate, ca. 1.2 mm long, pubescent, seemingly obtuse at the apex; floral bracts persistent, similar in colour and texture to the peduncle bracts, seemingly ovate, 2.8–3.1 mm long, acuminate; pedicel articulate with the calyx, whitish-green, subterete, 4.9–5.6 mm long, 3.0–3.5 mm thick, pubescent; bracteoles 2, located near the base and opposite, whitish-green, ovate-triangular, 1.0–1.9 mm × 0.8–1.1 mm, pubescent, apex acuminate, long-ciliate along the margins. *Flowers* pentamerous, reflexed, with pale green calyx and fuchsia red corolla, paler and whitish at the angles; calyx 6.0–7.5 mm × 5.2–6.4 mm, pubescent adaxially and sparsely pubescent abaxially; hypanthium obconical, 5-winged, 2.7–3.8 mm × 4.6–5.0 mm, truncate; limb spreading, open, 3.5–4.0 mm × 5.2–6.4 mm; lobes 5, deltate, 3.0–3.4 mm × 2.6–3.2 mm, slightly incurved at the apex, acuminate, sinuses acute. *Corolla* tubular, but narrowing distally and proximally, thick-carnose, bistratose, bluntly 5-angled, 4.3–4.5 cm long (including the lobes), 6.0 mm in diameter at the base, 5.0 mm in diameter at the throat, pubescent along the angles externally and in the internal apical half; lobes 5, spreading, narrowly linear-triangular, 17.5–20.9 mm × 1.8–2.3 mm, pubescent externally, glabrous internally, channelled and subverrucose internally, apex acuminate, slightly incurved. *Stamens* 10, with white filaments, golden yellow tubules and darker thecae, nearly equalling the corolla in overall length, 4.0–4.3 cm long; filaments connate forming a tubular staminal tube, 12.8–13.2 mm long, sparsely puberulent in both sides; anthers 3.5–3.6 cm long, each pair of thecae unequal, prognathous, 6.2–6.8 mm long, conspicuously granulose; tubules distinct, slightly unequal, seemingly connate in the distal 3/5, 2.8–3.0 cm long, glabrous, dehiscing by terminal pores, ca. 0.8 mm × 0.3 mm. *Style* pale green, exserted, 4.6–4.8 cm long, pilose, stigma truncated. *Fruits* not seen. (Fig. 4)

#### Diagnosis

The new species is most similar to *C.auriculatum*, but it is distinguished by the calyx limb without lobes at the base (vs. lobed), the smaller, acuminate calyx lobes, 3.0–3.4 mm × 2.6–3.2 mm (vs. acute, 9.0–10.0 mm × ca. 5.0 mm), not conspicuously nerved, pubescent (vs. prominently and reticulate nerved, weakly ciliate at the margin), the fuchsia red, pubescent corolla (vs. black-purple, weakly pilose), with longer lobes, 17.5–20.9 mm long (vs. 11.0–13.0 mm long) and the puberulent, longer filaments, 12.8–13.2 mm long (vs. glabrous, 6.0–7.0 mm long).

#### Etymology

The name of the new species is dedicated to its primary geographical distribution, the Province of Morona Santiago.

#### Distribution

*Ceratostemamoronasantiagoensis* is known only from the area surrounding the town of San Juan Bosco in south-eastern Ecuador. (Fig. 2).

#### Ecology

**Figure 5.**
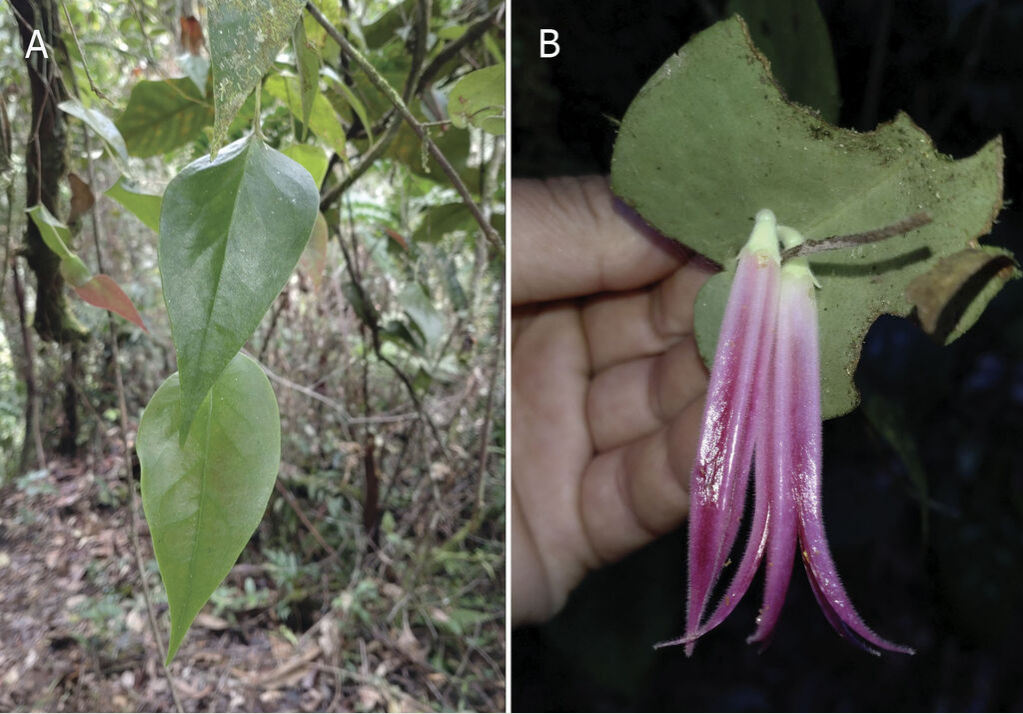
*Ceratostemamoronasantiagoensis* in situ. **A** Terminal branch; **B** Detail of the inflorescence seen from below.

*Ceratostemamoronasantiagoensis* is found as an epiphyte on tree trunks and branches near the canopy, showing no preference for a particular forest stratum. The species was seen at elevations between 1300 and 1600 m in the interior of mature primary forests with predominantly clayish soil conditions (Fig. 5). The type locality forest is predominantly represented by *Alchorneaglandulosa* Poepp., *Ingaoerstediana* Benth. and *Neeadivaricata* Poepp. & Endl. Specimens of *Ceratostemamoronasantiagoensis* were found sympatric with other flora such as *Dracontiumfuscopunctatum* Cornejo, Croat & G.Tello, Drymoniacf.coccinea (Aubl.) Wiehler, *Glossolomasubglabrum* J.L. Clark, *Pleurothallisvalvola* Luer & Hirtz and *Sobraliapardalina* Garay.

#### Conservation

*Ceratostemamoronasantiagoensis* was found near sites of forest clearing and agriculture, livestock farming and mining activities, consequently exhibiting the destructive, unsustainable nature of these operations within Ecuador. About ten individuals are known from four populations near San Juan Bosco in southern Morona Santiago Province, Ecuador. The extent of occurrence (EEO) calculated for the new species resulted in an area of 20 km^2^ with an area of occupancy (AAO) of 5.94 km^2.^ Given the aforementioned parameters, we recommend including the new species under the category of Critically Endangered (CR) according to the IUCN Criteria B1, B2, C2a(i) and D1 (IUCN 2024).

#### Taxon discussion

*Ceratostemamoronasantiagoensis* is most similar to *C.auriculatum* by the lianoid plants and the deeply cordate, pinnately nerved, glabrous leaves that are distinctly auriculate at the base. The basal lobes of the leaves are reflexed and do not conceal the flowers as seen in other members of this taxonomic lineage. This morphological trait extends to part of the inflorescence, where the reflexed leaves more or less obscure the inflorescence in *C.auriculatum* as is mentioned by Luteyn (1996). However, in *C.moronasantiagoensis* the inflorescences are likewise reflexed, thereby revealing the flowers. In consideration of the other members of the aforementioned “*auriculatum*” group, the leaves are generally folded at the base to fully conceal the flowers at all times. Further differences between *C.moronasantiagoensis* and *C.auriculatum* are the acuminate, narrower leaves that measure 4.5–6.9 cm wide (vs. short-acuminate, 5.5–9.6 cm wide); the shorter (4.9–5.6 mm long vs. 5.0–8.0 mm long), subterete pedicel (vs. bluntly-angled); the deltate calyx lobes (vs. ovate); the corolla being pubescent along the veinal angles (vs. glabrous or weakly pilose) and the lobes being incurved (vs. reflexed) upon anthesis (Luteyn 1996).

A dichotomous key and a table of morphological characters for the species of *Ceratostemaauriculatum* group are provided below (see Table 1).

## Identification Keys

### Key to the species of *Ceratostemaauriculatum* group

**Table:** 

1	Leaves ovate, pinnately nerved	2
–	Leaves suborbicular to orbicular-ovate	5
2	Leaves purple beneath, basal lobes parallel to each other	4
–	Leaves green beneath, basal lobes overlapping	3
3	Calyx limb basally lobulate, calyx lobes prominently and reticulate nerved, weakly ciliate at the margin	*C.auriculatum*
–	Calyx limb without lobes at the base, calyx lobes not conspicuously nerved, pubescent overall	***C.moronasantiagoensis***
4	Leaves short-pilose, calyx and corolla terete to 5-angled, calyx lobes > 4 mm long	*C.pendens*
–	Leaves glabrous, calyx and corolla 5-winged, calyx lobes < 4 mm long	*C.ingridportillae*
5	Twigs lianoid, leaves floccose-tomentose abaxially, calyx lobes > 3 mm long	*C.macbrydiorum*
–	Twigs filiform, leaves glabrous abaxially, calyx lobes < 3 mm long	6
6	Leaves > 9 cm broad, floral parts glabrous, calyx and corolla winged, calyx lobes < 1 mm long	*C.cutucuense*
–	Leaves < 9 cm broad, floral parts puberulous, calyx and corolla not winged, lobes > 1 mm long	***C.gearyana***

## Supplementary Material

XML Treatment for Ceratostema gearyana

XML Treatment for Ceratostema moronasantiagoensis

## Figures and Tables

**Table 1.** **Table 1.** Morphological comparison of the species of *Ceratostemaauriculatum* group. AUR: *C.auriculatum*; CUT: *C.cutucuensis*; GEA: *C.gearyana*; ING: *C.ingridportillae*; MAC: *C.macbrydiorum*; MOR: *C.moronasantiagoensis*; PEN: *C.pendens*. References taken from: (1, 2, 5) Luteyn (1996); (3, 6) presented herein; (4) Doucette et al. (2025c); and (7) Luteyn (2005).

	**AUR (1)**	**CUT (2)**	**GEA (3)**	**ING (4)**	**MAC (5)**	**MOR (6)**	**PEN (7)**
**Twig pubescence**	Glabrous	Glabrous	Puberulous	Glabrous	Densely hirsute	Pilose	Short-pilose
**Leaf**							
Posture	Amplexicaul, flat to somewhat incurved, thus hiding flowers	Amplexicaul, flat	Spirally arranged, pendulous	Spiral to distichous, sessile	Amplexicaul, flat	Spirally arranged, pendulous to descending	Amplexicaul, involute
Apex	Acuminate	Cuspidate to acute	Shortly acuminate to acuminate	Acuminate	Short-acuminate	Acuminate to long–acuminate	Acuminate
Pubescence	Glabrous	Glabrous	Glabrous adaxially, with very few sparse hairs at the basal margin abaxially	Glabrous	Pilose (glabrate adaxially)	Glabrous	Pilose both surfaces
Venation	Pinnate	5–plinerved	7–9 plinerved	Pinnate to weakly plinerved	5-plinerved	Pinnately nerved with 3–4 lateral nerves per side	Pinnate to weakly plinerved
**Calyx**							
Overall length (mm)	12.0–14.0	8.5–10.0	7.2–8.9	8.1–8.5	ca.28	6.0–7.5	8.0–9.0
Tube cross–section	5–winged	5–winged	Obscurely pentagonal	5–winged	5-winged	5–winged	Terete to 5-angled
Tube length (mm)	3.0–4.5	6.5–7.0	5.5–6.3	5.8–6.0	6.0	2.7–3.8	2.7–3.5
Lobe length (mm)	9.0–10.0	<0.5	2.0–2.7	2.3–2.5	ca. 21 mm	3.0–3.4	4.8-5.0
Lobe glands	Glandular–fimbriate	Eglandular	Eglandular	Eglandular	Eglandular	Eglandular	Eglandular
**Pedicel**							
Length (mm)	5.0–8.0	12.0–13.0	7.6–8.4	6.0	12.0–13.0	4.9–5.6	5.0–6.0
Pubescence	Pilose	Glabrous	Puberulous	Glabrous	Glabrous	Pubescent	Pilose
**Corolla**							
Length (mm)	45.0–47.0	ca. 50.0	42.0–44.0	31.2–33.0	Not mentioned	43.0–45.0	45.0–48.0
Cross–section	Terete to bluntly 5–angled	5–winged over entire length	Bluntly 5–angled along its length externally	5–winged	Not mentioned	Bluntly 5–angled	Terete to bluntly 5–angled
Pubescence	Glabrous to sparsely pilose along angles	Short–pilose	Puberulent along its external length, puberulous in the internal apical half	Glabrous	Not mentioned	Pubescent externally, glabrous internally	Glabrous
**Stamens**							
Length (mm)	ca. 43.0	ca. 50.0	36.0–40.0	ca. 43.0–45.2	Not mentioned	40.0–43.0	45.0–46.5
Filaments	Connate	Connate	Connate	Connate	Not mentioned	Connate	Connate
